# On Dentition, and Some Coincident Disorders

**Published:** 1834-04-01

**Authors:** 


*On Dentition, and some coincident Disorders.* By J. Ashburner, m.d.—London, 1834. 12mo. pp. 235.

This is a reprint, with additions, of some papers published in the Medical Gazette; and we must confess that they looked better in the close columns of our excellent contemporary than in their present form; for, though we are not among those who think that doctors or their books should come out in black, still we disapprove of a grave medical treatise looking, in externals at least, like a volume of fairy tales. However, let that pass.

Dr. Ashburner supposes that constitutional diseases very frequently arise from irregularities in the second set of teeth: the wise teeth cannot cut their way into the world, or the incisors overlap each other, and hysteria, epilepsy, &c. are the consequences. This is Dr. Ashburner's hobby, and he rides it to death. In the last case in the book (p. 232,) a young lady, disappointed in love, became first of all hysterical, and afterwards epileptic. Here the wise teeth, the incisors, and the bicuspides, were all in fault. Our author, however, in this case, did not attempt to set the teeth to rights, but very judiciously advised marriage: the prescription was taken, and the patient recovered completely.

We should have called the general run of Dr. Ashburner's cases extremely rare ones, but he meets with them by shoals. We gave several in the Collectanea of our last number, and shall therefore content ourselves with quoting one of the most remarkable.

"An eminent physician, who had practised in a large provincial city, was passing through London. I met him in Regent street, and the suddeness of my approach threw him into a state of obliviousness. He did not venture out without a companion, and the lady who was walking with him hinted that I had better call on him the next day. When I saw him again, he gave me a very clear account of the mode in which he was attacked. The fits of oblivion occurring sometimes twice in the course of the day, and being uncertain as to the periods of their accession, he was not able to trust himself out of his house without either a servant or a companion. He was at this time fifty-eight years of age, and had six years before been obliged to relinquish a lucrative practice, from occasionally not being able to recollect even the faces of his patients when they appeared before him. He went away to travel on the continent, and journeying from place to place in Italy, where the classic ground ought to have raised emotions of great delight in a healthy mind, so well educated as this gentleman's, he had constantly to regret that a fit of oblivion attacked him when he was engaged in viewing scenes which were of deep interest to his fellowtravellers. I remarked a curious arrangement of the inferior incisors when his mouth opened: there were only three in a space which ought to be occupied by four teeth. I learned that one had been removed nearly eight years before. But if one had been extracted the room was now filled up, so that a pressure from the back part of the jaw had obliterated the vacant space, and caused a complete approximation of the edges of the two incisors left apart from each other. On looking into the mouth, it was found that the last molares, above and below, had never been cut, that in the upper jaw the vis-a-tergo maxillæ had played sad havoc among the teeth: a few stumps were left, the immediate removal of which I advised. I cut away for him myself some cartilaginous obstructions to the progress of his wise teeth, which appeared, from long pressure, to have suffered in their integrity quite as much as the other teeth. He remained in London about a fortnight after, and told me that he was so much relieved of his oblivion fits as to be able to walk to my house without any want of confidence in himself: he required no companion." (P. 230.)

